# High Expression of MRE11A Is Associated with Shorter Survival and a Higher Risk of Death in CRC Patients

**DOI:** 10.3390/genes14061270

**Published:** 2023-06-15

**Authors:** Daniel de Barcellos Azambuja, Helena de Castro e Gloria, Gabriel e Silva Montenegro, Antonio Nocchi Kalil, Jean-Sébastien Hoffmann, Natalia Motta Leguisamo, Jenifer Saffi

**Affiliations:** 1 Laboratório de Genética Toxicológica, Universidade Federal de Ciências da Saúde de Porto Alegre (UFCSPA), Porto Alegre 90050-170, RS, Brazil; azambujadb@gmail.com (D.d.B.A.); helenadecastroegloria@gmail.com (H.d.C.e.G.); ankalil@ufcspa.edu.br (A.N.K.); nmleguisamo@gmail.com (N.M.L.); 2 Hospital Santa Rita, Irmandade Santa Casa de Misericórdia de Porto Alegre (ISCMPA), Porto Alegre 90020-090, RS, Brazil; 3 Laboratoire d’Excellence Toulouse Cancer, Laboratoire de Pathologie, CHU Toulouse, Institut Universitaire du Cancer-Toulouse, Oncopole, 1 Avenue Irène-Joliot-Curie, CEDEX, 31059 Toulouse, France; jean-sebastien.hoffmann@inserm.fr

**Keywords:** colorectal cancer, homologous recombination, prognosis, MRE11

## Abstract

Background: Homologous recombination repair (HR) is the most accurate repair pathway for double-strand breaks and replication fork disruption that is capable of faithfully restoring the original nucleotide sequence of the broken DNA. The deficiency of this mechanism is a frequent event in tumorigenesis. Therapies that exploit defects in HR have been explored essentially in breast, ovarian, pancreatic, and prostate cancers, but poorly in colorectal cancers (CRC), although CRC ranks second in mortality worldwide. Methods: Tumor specimens and matched healthy tissues from 63 patients with CRC were assessed for gene expression of key HR components and mismatch repair (MMR) status, which correlated with clinicopathological features, progression-free survival, and overall survival (OS). Results: Enhanced expression of MRE11 homolog (*MRE11A*), the gene encoding a key molecular actor for resection, is significantly overexpressed in CRC, is associated with the occurrence of primary tumors, particularly T3-T4, and is found in more than 90% of the right-side of CRC, the location with the worst prognosis. Importantly, we also found that high *MRE11A* transcript abundance is associated with 16.7 months shorter OS and a 3.5 higher risk of death. Conclusion: Monitoring of MRE11 expression could be used both as a predictor of outcome and as a marker to select CRC patients for treatments thus far adapted for HR-deficient cancers.

## 1. Introduction

Among the multiple DNA lesions that occur recurrently in human cells, the DNA double-strand breaks (DSBs), which result from direct environmental chemical attacks or the collapse of stalled replication forks, constitute the greatest threat to genomic stability. Several conserved and mechanistically distinct pathways of DSB repair have evolved to repair DNA damage. These include homologous recombination (HR), non-homologous end-joining (NHEJ), alternative end-joining, and single-strand annealing [1,2]. While the NHEJ, alternative end-joining, and single-strand annealing pathways often cause deletion or insertion of several nucleotides and can trigger chromosome translocations, HR is the most accurate DSB repair mechanism capable of faithfully restoring the original configuration of the broken DNA molecule. HR is the most conservative process that uses a sister chromatid as a template to accurately fix the DSBs. In contrast to NHEJ, the first essential step of HR is DNA resection at the break termini, which is controlled by breast cancer-associated gene 1 (*BRCA1*) in a cell cycle-dependent manner by restricting access of the NHEJ factor tumor protein p53 binding protein 1 (TP53BP1) to the ends of DSBs, thereby permitting the generation of recessed DNA with 3′overhangs [3]. Such 5′ to 3′ end resection, which is performed by the highly conserved MRN complex comprising the MRE11 homolog (MRE11), RAD50 double-strand break repair protein (RAD50), and nibrin (NBN) factors in conjunction with RB binding protein 8 (RBBP8), is of paramount importance for the subsequent HR steps, which consist of the binding of the partner and localizer of BRCA2 (PALB2) to BRCA1, which in turn directs breast cancer-associated gene 2 (*BRCA2*) to promote RAD51 recombinase (RAD51) filament formation on the recessed DSB. This is a critical step necessary for homology searching, strand invasion, and repair synthesis [4].

The deficiency of such a conservative repair pathway is a frequent event in tumorigenesis, which provides a selective growth advantage to tumor cells as it results in replicative stress, genetic instability, and enhanced mutation rates, which are the driving forces for tumor evolution. Multiple hereditary or somatic cancers are deficient in HR. This has been particularly well described in tumors with germline mutations in BRCA1/2, such as breast and ovarian cancers [5]. Homologous recombination deficiency (HRD) has been documented to be associated with higher sensitivity to alkylating or platinum-based agents due to the generation of non-processed and highly toxic DSBs [6,7]. Most importantly, the use of poly(ADP-ribose) polymerase (PARP) inhibitors (PARPi) has been approved by the US Food and Drug Administration and the European Medicines Agency to implement these classical drugs to treat ovarian and breast HR-deficient tumors through a synthetic lethality concept [8].

Generally, therapies that exploit defects in HR have been explored essentially in breast, ovarian, pancreatic, and prostate cancers, but poorly in colorectal cancers (CRC), although it is a commonly diagnosed malignant neoplasm that ranks third among all cancers in terms of incidence and second in mortality worldwide [9]. Discrepancies within TNM (Tumor, node, metastasis) stages of CRC patients have been widely reported, primarily due to contradictions in the survivorship of patients harboring stages IIB/C or IIIA disease [10,11,12]. Moreover, the therapeutic repertoire for CRC remains limited, with few targeted agents and companion diagnostics endorsed for clinical use [13]. Despite the efforts to identify a molecular signature to improve prognosis prediction and CRC patient selection [14,15,16], a biomarker-enriched classification has not yet reached clinical translation. Recent data have demonstrated that 10–30% of CRC harbor somatic mutations in genes involved in the DNA damage response (DDR), which could explain some aspects of resistance to therapy and a poor prognosis [17,18]. Among these, germline pathogenic variants of BRCA1, ATM, and PALB2 have been associated with a greater CRC risk, and up to 15% of all CRC present germline or somatic alterations in HR genes [19]. Moreover, it has been suggested that a subset of CRC patients harbors mutations in HR genes, including ATM serine/threonine kinase (ATM), BRCA1/2, MRE11A, FA complementation group C (FANCC), NBS1, and PALB2 [20].

In this study, we further explored the link between HR genes and CRC by examining the prognostic significance of the expression of genes encoding proteins involved in the key steps of HR in a series of CRC patients.

## 2. Materials and Methods

### 2.1. Patients and Tissue Collection

This study was conducted at the Santa Rita Hospital (Irmandade Santa Casa de Misericórdia de Porto Alegre, Porto Alegre, Brazil). Patients with histologically confirmed adenocarcinoma of the colon and rectum who were admitted for colorectal surgery were eligible. Patients with non-primary CRC, familial adenomatous polyposis, or inflammatory bowel disease were excluded. The variables of interest included age, gender, date of surgical resection, primary location, tumor grade, laterality, vascular invasion, perineural invasion, preoperative carcinoembryonic antigen (CEA) levels, and treatment. Histopathological data (such as tumor subtype, depth of invasion, lymph node and/or metastasis distance, and staging) were also extracted from the pathological reports. The pathological TNM stage was used as the staging scale for prognosis [21]. The outcomes of interest were disease-free survival (DFS) and overall survival (OS). DFS was defined as the time from diagnosis to the first recurrence. OS was defined as the time from diagnosis to the last follow-up or death.

A total of 63 primary colorectal neoplasms from sporadic CRC patients were collected between March 2013 and July 2016. These patients were followed from the date of surgical tumor resection until December 2022. At least two fresh tissue samples were collected from each patient’s surgical specimen: full-thickness colorectal tumor and adjacent normal colon or rectal tissue at least 10 cm away from the largest tumor. Neoplastic and healthy tissues were immediately embedded in RNAlater™ stabilization solution (Invitrogen) for 24 h and then frozen for subsequent analyses. The CRC series data were collected prospectively; patients were informed and signed a written consensus for collecting data and biological samples. The ethical committees of the participating institutions approved the study, and written informed consent was obtained from the patients before study enrollment. The research conformed to the Helsinki Declaration and was approved by the Regional Committee for Medical and Health Research Ethics (CAAE: 58299916.3.3001.5345).

### 2.2. Gene Expression Analysis and HRD Assessment

Total RNA was extracted using RNeasy Mini kit (Qiagen, São Paulo, Brasil), and cDNA synthesis was performed in a 20-µL reaction with 1 µg of total RNA using RT2 FirstStrand Kit (Qiagen) according to the manufacturer’s instructions. Quantitative RT-PCR was performed in duplicates by RT2 Profiler™ PCR Array (SABiosciences/Qiagen) using RT2 SYBR Green qPCR Mastermix (Qiagen) and StepOne Plus apparatus (Applied Biosystems™). Custom RT2 Profiler PCR Array (#CLAH-32033-9619-6, Qiagen) included MRE11 homolog (MRE11A), RAD50 double-strand break repair protein (RAD50), nibrin (NBN), breast cancer-associated gene 1 (BRCA1), BRCA1-associated RING domain 1 (BARD1), RB binding protein 8 (RBBP8), and the binding of partner and localizer of BRCA2 (PALB2) genes. Threshold cycle (Ct) values for each duplicate were normalized to the geometric average of the housekeeping genes ACTB (β-actin) and PPIA (peptidylprolyl isomerase A). An average of the resulting values was calculated to obtain ΔCt values for biological replicates. Relative mRNA (ratio between ΔCt in neoplastic tissue and ΔCt in healthy tissue) was calculated, and a log2 transformation was applied. Gene expression was dichotomized into “high” and “low” according to the median fold change of each HRR gene (expression ≤ median = low; expression > median = high). We also classified a gene with a fold change > −2.0 as “deficient”, i.e., the gene expression was significantly reduced in the tumor tissue [22].

### 2.3. Investigation of Microsatellite Instability

The first method for microsatellite instability (MSI) testing is MMR immunohistochemistry (IHC). IHC is a widely available laboratory test that utilizes antibodies against the following four MMR proteins: MLH1, MSH2, MSH6, and PMS2. This test is considered the gold standard for mismatch repair deficiency, which causes microsatellite instability [23]. Thus, the status of MSI was evaluated using immunohistochemistry. Briefly, 3-µm thick FFPE tissue sections were deparaffinized in xylene, rehydrated in graded alcohols, washed in double-distilled water, and pretreated with DAKO solution (EnVision FLEX Target Retrieval Solution, High pH) at 97 °C. The slides were then incubated with primary monoclonal antibodies against MLH1 (clone ES05, DAKO), PMS2 (clone EP51, DAKO), MSH2 (clone FE11, DAKO), MSH6 (clone EP49, DAKO), and EnVision FLEX+ Mouse (LINKER) for 30 min. The analysis was performed on the automated platform Autostainer Link 48 (Dako, Carpinteria, CA, USA) according to the manufacturer’s instructions. The antigen–antibody reaction was inspected using the EnVision FLEX kit with diaminobenzidine as the chromogen; slides were counterstained with hematoxylin and then covered. MMR protein expression was categorized as retained (i.e., proficient MMR; pMMR) when a moderate to strong nuclear protein expression was detected in tumor cells as well as in internal controls, and lost (i.e., deficient MMR; dMMR) when a complete loss of nuclear expression in tumor cells was observed but retained in normal cells.

### 2.4. Inflammation-Related Peripheral Blood Measurements

The absolute counts of circulating neutrophils, lymphocytes, and platelets are associated with inflammatory responses, which are key factors in recognizing pathways for tumorigenesis and growth [24]. Thus, we calculated the neutrophil-to-lymphocyte ratio (NLR), lymphocyte-to-monocyte ratio (LMR), and platelet-to-lymphocyte ratio (PLR) from the absolute counts of pretreatment peripheral blood tests. Subgroups were divided using the median expression value (expression ≤ median = low; expression > median = high).

### 2.5. Statistical Analyses

Statistical analyses were performed using IBM SPSS Statistics 29.0. The OS and DFS rates were estimated using the Kaplan–Meier method. The log-rank test was used to assess significant differences between subgroups using univariate analysis. To investigate independent prognostic factors for OS and DFS, factors with a *p* < 0.2 in univariate analyses were entered into multivariate analysis. The Cox proportional hazards regression model was used to identify the factors that were independently associated with OS. Pearson’s chi-squared test and Fisher’s exact test were used to evaluate distributions of categorical variables. The *p*-values less than 0.05 were considered statistically significant.

## 3. Results

### 3.1. Characteristics of CRC Patients

A total of 63 patients with histologically confirmed sporadic colorectal adenocarcinoma who underwent surgical tumor resection between March 2013 and July 2016 were included. The clinicopathological features are summarized in Table 1. Forty patients (63.5%) were male, the mean age of CRC diagnosis was 64 years old (range 35–87), and 52% were 65 years old or less. Forty patients (64.0%) presented with a primary tumor location in the colon. Among them, twenty-seven patients (67%) harbored left-sided CRC. Eight patients (13%) presented with metastatic disease at diagnosis. Preoperative CEA < 5 ng/dL was observed in 45 patients (71%), and 40 (63%) presented with moderate or high tumor grade. MMR deficiency was present in 13% (8 patients) of the primary tumors. Representative patterns of MMR protein staining are shown in Supplementary Figure S1. At the data analysis time, with a mean follow-up of 61 months (range 6–113), 18 patients (29%) had reported disease progression, and 34 (54%) were still alive.

### 3.2. Alteration of HR Gene Expression in CRC

**Figure 1:**
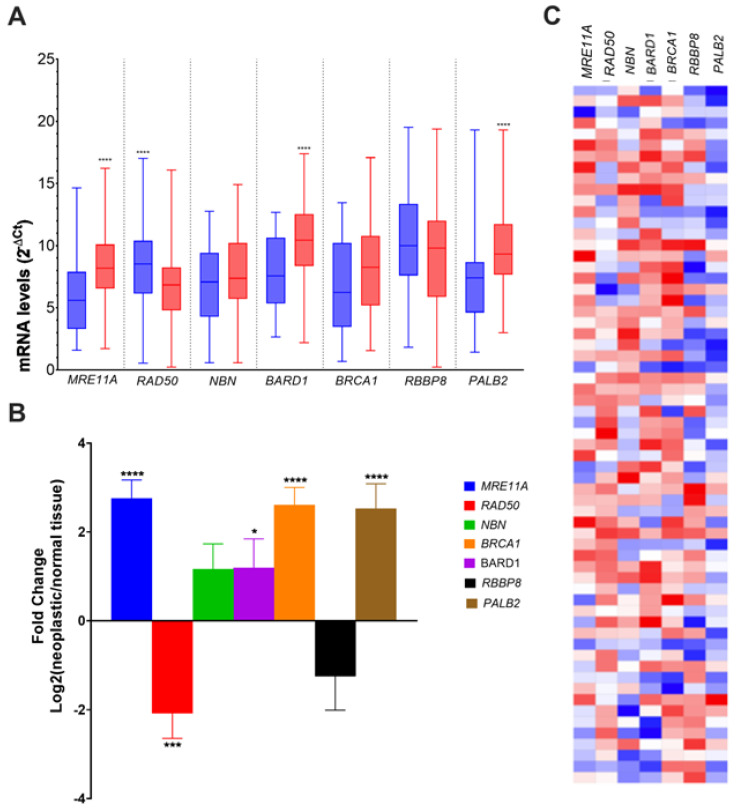
Changes in the gene expression of representative HR components in neoplastic colorectal specimens and matched healthy tissues. (**A**) mRNA levels (mean ± SEM). Blue boxplots represent normal tissues and red boxplots represent colorectal tumors. (**B**) Fold change between neoplastic and normal tissues (mean ± SEM). (**C**) Heatmap showing the individual gene expression fold changes of key HRR components in colorectal tumors. Blue represents the expression ≤ median = low; red represents the expression > median = high; gene expression means between normal and neoplastic tissues and fold changes were compared using Mann–Whitney’s test and Wilcoxon test, respectively; * *p* < 0.05; *** *p* < 0.001; **** *p* < 0.0001.

**Figure 2:**
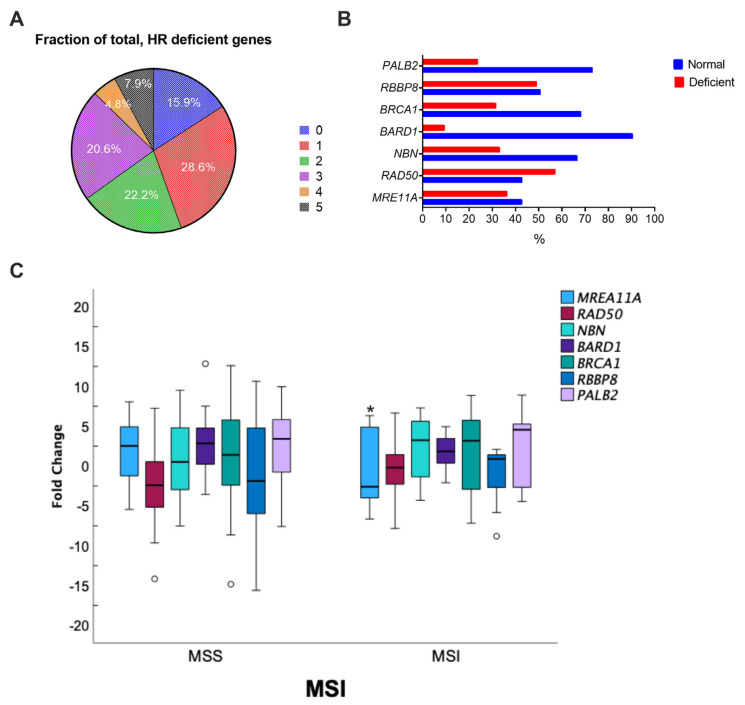
Characterization of HR profiles in CRC patients. (**A**) Proportions of patients according to the number of deficient HR genes in tumor samples (fold change > −2.0); (**B**) proportions of each HR gene according to normal or deficient expression; (**C**) boxplots of HR gene fold change means according to MMR status. T-test for equality means. * *p* (two-sided) < 0.05; the circles represent the outliers.

The mRNA levels for key actors involved in important steps of HR, *MRE11A*, *RAD50*, *NBN*, *BARD1*, *BRCA1*, *RBBP8*, and *PALB2* were quantified using qPCR in 63 pairs of primary sporadic colorectal tumors and matched adjacent tissues. *MRE11A* (*p* < 0.001), *BARD1* (*p* < 0.001), and *PALB2* (*p* < 0.001) were significantly overexpressed (Figure 1A), with a significant mean fold induction of 3.28 (*p* < 0.0001) for *MRE11A*, 2.83 (*p* < 0.0001) for *BARD1*, and 2.09 (*p* = 0.373) for *PALB2* (Figure 1B). In contrast, *RAD50* (*p* < 0.001) mRNA levels were significantly reduced (Figure 1A) in comparison to healthy tissues, with a mean −2.15-fold decrease (*p* < 0.001) (Figure 1B). For *NBN*, *BRCA1*, and *RBBP8*, no difference in gene expression between tumoral and adjacent normal tissues was observed (Figure 1B). When we analyzed the proportion of CRC patients according to the number of reduced HR gene expression in tumor samples (fold change > −2.0), we found that about 50% of the patients harbored one or two altered gene expression, 8% of the patients showed a deficiency of expression for five HR genes concomitantly, and 16% of the patients had no deficiency in HR gene expression (Figure 2A). We observed that *RAD50* expression was the most affected among the HR genes (57.1%), whereas *BARD1* was deficient in only 9.5% of the patients. Interestingly, when the modified HR gene expression was explored according to MSI status, only *MRE11A* was significantly affected in MSI colorectal tumors (−2.43, IC 95% −4.96–0.10; *p* = 0.039) (Figure 2C). More details on the fold change difference and T-test results for equality of means are given in Supplementary Table S1.

### 3.3. Associations between HR Gene Expression and Clinicopathological Features in CRC

Next, we investigated the possible associations between the clinicopathological features of CRC patients and the gene expression of *MRE11A*, *RAD50*, *NBN*, *BRCA1*, *RBBP8*, and *PALB2* (Table 2). A low expression of *RAD50* was found in 68% of left-sided CRC (*p* = 0.024) and 75% (*p* = 0.044) of tumors with no perineural invasion. A low expression of *NBN* was found in 75% (*p* = 0.044) of tumors in the initial stages, T1-T2. *RBBP8* was not associated with any clinicopathological feature. A high expression of *BARD1* was found in young patients (65 years old or less) (*p* = 0.046) and in patients with low TNM stage (*p* = 0.044), TNM II-III. A normal or high expression of *BRCA1* was correlated with patients with no regional lymph nodes affected (*p* = 0.040). For *PALB2*, we found that a relatively active high expression of this gene was associated with age at diagnosis (*p* = 0.022), absence of nodal metastasis (*p* = 0.002), TNM I-II (*p* = 0.001), and absence of lymphovascular invasion (*p* = 0.015). Finally, we found that a high expression of *MRE11A* was associated with the occurrence of primary tumors in the colon (*p* = 0.008), and 92% of these cases with high *MRE11A* abundance were found on the right side (*p* = 0.042) of the colon, which corresponds to the worst prognosis. Enhanced *MRE11A* expression was also associated with T3-T4 tumors (*p* = 0.04). Collectively, these data support the fact that only *MRE11A* high expression shows a critical correlation with the most pejorative features of CRC.

### 3.4. Analysis of Inflammatory Features in CRC Patients and Crosstalks with HR

**Figure 3:**
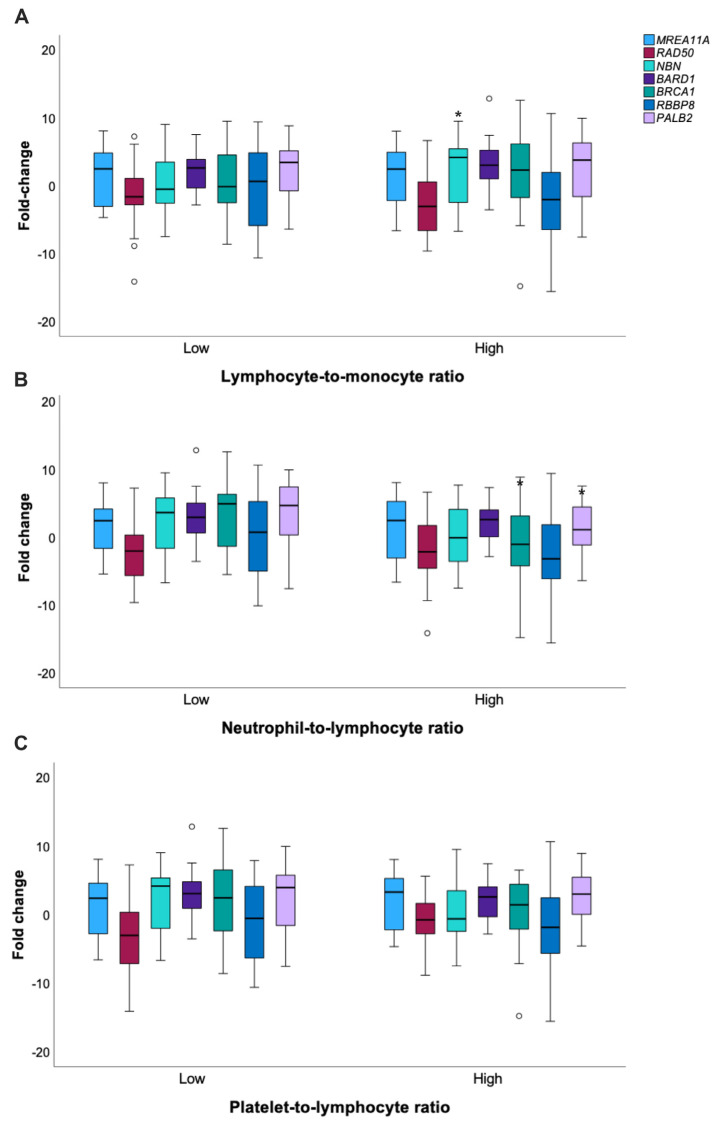
Fold changes of HR representative genes according to composite inflammatory blood indexes. T-test for equality means. * *p* < 0.05; the circles represent the outliers (**A**). Lymphocyte-to-monocyte ratio; (**B**). Neutrophil-to-Lymphocyte ratio; (**C**). Platelet-to-lymphocyte ratio.

Considering that systemic inflammatory factors also promote cancer growth and metastasis (34) and that crosstalk between DNA damage response and inflammation has been evidenced (35), we investigated the possible associations of perioperative absolute counts of lymphocytes, neutrophils, monocytes, and platelets and their derived inflammation-based indexes with HR gene expression profiles (Figure 3). The median NLR, LMR, and PLR values (2.1, 2.7, and 131.3, respectively) were used as cut-offs to determine subgroups with low (≤median) or high (>median) inflammatory blood indexes. The mean NBN fold change was significantly higher in patients harboring a high LMR (2. vs. 18 0.11 *p* = 0.039). In contrast, both BRCA1 and PALB2 fold-induction were inversely correlated with NLR, i.e., these genes were found to be overexpressed in patients who presented low neutrophil-to-lymphocyte ratios (Supplementary Table S2).

Associations between inflammatory blood indexes and clinicopathological features of CRC patients were also explored (Supplementary Table S3). A high LMR was observed in 63% of the patients younger than 65 years old with a diagnosis (*p* = 0.029), whereas a low LMR was present in 70% of patients with tumors <3 cm (*p* = 0.043) and perineural invasion (*p* = 0.044). The NLR was associated with tumor size (*p* = 0.021), tumor invasive depth (*p* = 0.008), and perineural invasion (*p* = 0.040). Almost 80% and 70% of tumors diagnosed with more than 3 cm and perineural invasion, respectively, occurred in patients with NLR > 2.1. Conversely, 81.3% of the patients with a low NLR were staged as pT1 or pT2.

### 3.5. High MRE11A Expression Is Associated with Poor Survival in CRC

**Figure 4:**
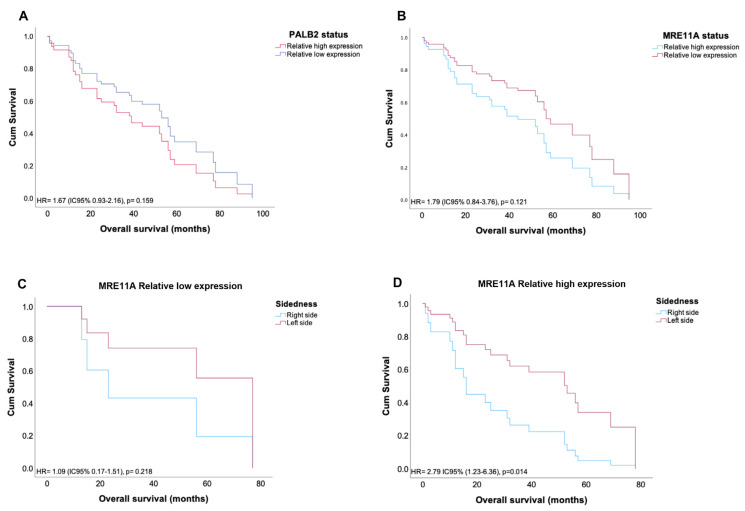
Kaplan–Meier plots of Cox regression for progression free survival of CRC patients. Survival curves of (**A**) PALB2; (**B**) MRE11A; (**C**) MRE11A, relative low expression adjusted for sidedness; and (**D**) MRE11A, relative high expression adjusted for sidedness.

Using univariate and multivariate analyses, we explored the characteristics of the CRC patient cohort, which influenced the prognostic value of DFS and OS. The univariate analysis of DFS (Table 3) identified the following potential independent prognostic features: age (*p* = 0.148), CEA levels at diagnosis (*p* = 0.087), sidedness (*p* = 0.098), lymphovascular invasion (*p* = 0.029), TNM stage (*p* = 0.035), MMR status (*p* = 0.140), and MRE11A (*p* = 0.183). Cox regression tests of coefficients did not confirm any of these features as independent prognostic factors for composite outcome disease progression in sporadic CRC patients. Mean OS was 51.9 months IC 95% (44.8–58.9). The univariate analysis of molecular and clinicopathological variables identified tumor location (*p* = 0.201), sidedness (*p* < 0.001), TNM stage (*p* = 0.076), MMR status (*p* = 0.157), MRE11A status (*p* = 0.121), and PALB2 status (*p* = 0.259) as candidates to independently predict the risk of death in CRC patients (Table 4). The test results of the model reached statistical significance (*p* = 0.007). Cox regression confirmed the independent prognostic value for sidedness (HR = 4.57 IC95% [1.55–13.49], *p* = 0.006), MRE11A status (HR = 3.11 IC95% [1.64–15.08], *p* = 0.046), and PALB2 status (HR 2.06 IC95% [1.67–17.63], *p* = 0.044). Kaplan–Meier plots for OS adjusted by MRE11A and PALB2 status are shown in Figure 4. MRE11A status (HR = 2.80 IC95% [1.23–6.36], *p* = 0.016) and PALB2 status (HR = 3.33 IC95% [1.44–7.70], *p* = 0.005) adjusted for sidedness are presented as independent prognostic factors for overall survival in patients with CR.

## 4. Discussion

HR is a multistep pathway that starts with the complex formation of BRCA1 and BARD1, leading to its removal from the DSB of 53BP1-RIF1, a complex known to recognize and protect DSB, and access of nucleases that extensively resect the 5′ DNA ends. This resection step generates single-stranded DNA (ssDNA) several kilobases long on each side of the break. This resection step is essential, as ssDNA hybridizes with complementary sequences on a sister chromatid and is then extended to allow efficient and accurate repair. The MRN complex is involved in this resection step and includes the critical nuclease factor MRE11 (meiotic recombination 11), together with RAD50 and NBS1 (Nijmegen breakage syndrome 1). The C-terminal binding protein and interacting protein (CtIP) contribute to this process by stimulating the exonuclease activity of MRE11. After CtIP-stimulated DNA resection, the stretches of ssDNA are rapidly coated with RPA, protecting them from degradation. BRCA2 associated with PALB2 then promotes the loading of the RAD51 recombinase, replacing RPA and the RAD51 molecules to form nucleoprotein filaments, which catalyze invasion and a homology search of the sister chromatid. Variants in key genes of HR, such as BRCA1, BRCA2, PALB2, BARD1, RAD51C, and RAD51D, which alter their function and/or their expression, have a significant association with breast cancer risk [25,26]. The variants of some of these genes are also associated with ovarian, pancreatic, and prostate cancer. In CRC, HR alterations have also been found in up to 20% of CRC cases [17,19,20,27,28,29]. Most importantly, none of these gene mutations were found in the encoding factors that participate in the resection step of HR.

In this study, we report alterations in HR gene expression in CRC, including BRCA1, BARD1, and PALB2, but also in genes involved in the resection, such as RAD50 and MRE11. Notably, we report that for one of them, MRE11, its relatively high expression was associated with the pejorative features of CRC, i.e., right-sided colon tumors, increased tumor invasive depth, higher tumor grade, MMR deficiency, and poorer overall survival. Interestingly, high expression of Mre11 was previously shown to be associated with poor prognosis in rectal cancer patients treated with radiotherapy and in patients with right-side localized CRC [30]. In colon cancer cohort (TCGA-COAD) data analysis, a tendency of correlation was observed between high MRE11 and worse survival probability. Mechanistically, why does overexpression of MRE11 lead to such unfavorable outcomes for CRC patients? In this cohort, more than 90% of the patients with right-sided CRC also harbored MRE11A upregulation. Right-sided CRC has a higher incidence of dMMR/MSI and an increased tumor mutational burden, which favors the local recruitment of tumor-infiltrating lymphocytes. While MRE11 has been described as disrupted in over 60% of dMMR/MSI CRC, it has been suggested that MRE11 expression is strongly correlated with the activation of the immune response, as evidenced by higher levels of tumor-infiltrating inflammatory cells [31]. This could be explained by the short pieces of DNA generated in the cytosol by enhanced ssDNA degradation, which in turn can trigger strong innate immune responses, including the production of type I interferons (IFNs) and other inflammatory cytokines via binding to these DNA fragments of cGAS, the major sensor for cytosolic DNA [32]. Thus, a high level of Mre11 could be a potential predictive biomarker for response to immunotherapy in CRC. Furthermore, a high abundance of Mre11 would interfere and compete with the normal process of HR and lead to exacerbated resection of DSB, creating excessive ssDNA and, in turn, excessive DSB, a source of chromosome instability and consequently enhanced cell variability, explaining why excess Mre11 is associated with poor prognosis. Such high resection levels by overexpressed MRE11A may sensitize CRC cells to agents that interfere with the progression of the replication forks, including PARPi, whose toxicity has been recently associated with the presence of gap DNA at the forks [33], as these agents would exacerbate ssDNA gap accumulation.

During HR, the partner and localizer of BRCA2 (PALB2) interacts with BRCA1 and BRCA2 and plays a crucial role in the repair of DSB. While BRCA1 recruits PALB2 at the sites of DNA breakage, PALB2 stabilizes BRCA2 during the formation of the RAD51 nucleoprotein filament. Mutations in the PALB2 gene have been associated with increased cancer risk, notably for breast cancer; however, the importance of its expression has been poorly explored. In this study, we found that, similarly to MRE11, PALB2 was significantly overexpressed in CRC tumors compared to the adjacent normal tissues. Furthermore, elevated PALB2 expression was associated with poorer overall survival, suggesting that PALB2 expression levels may also serve as a novel prognostic factor for colorectal cancer patients, similar to MRE11. It is worth noting that concurrent overexpression of two or three genes is a rare occurrence in patients. This might be attributed to the fact that this situation could completely impair the HR process, potentially leading to lethality in the tumor cells. In such cases, there would be no possibility to rescue the repair of DSB, as neither the reversion mutation of BRCA nor the alternative MMEJ pathway would be functional or able to compensate.

Another crucial aspect that should be considered for CRC prognosis is the primary tumor sidedness. Recent studies have demonstrated that patients with right-sided tumors have a worse prognosis [34,35]. In a systematic review and meta-analysis published by Petrelli et al., it was demonstrated that laterality has prognostic value independent of stage, race, and adjuvant chemotherapy [36]. We have identified that more than 90% of the cases with high expression of MRE11A were located on the right side of the colon. Additionally, in Kaplan–Meier analyses, we identified that a high expression of MRE11A in the right-sided CRC was significantly correlated with worse OS. Taken together, these results suggest that MRE11A overexpression may have a prognostic value for poor CRC outcomes.

Overall, our study highlights that among the key HR genes that are differentially expressed in our cohort of patients with sporadic CRC, only MRE11A upregulation holds a strong clinical value in terms of tumor aggressiveness, sidedness, and survival prediction. These results warrant further validation in larger CRC cohorts, and it would be important to explore in future research whether Mre11 high-expressing tumors could be potential candidates for immunotherapy or agents that target DNA replication.

## Figures and Tables

**Table 1:** Clinicopathological features of CRC patients.

Variable	n (%)	*p* Value
Total cases	63 (100)	
Age (years; mean ± SD)	64 ± 11	
Age (years)		0.705
≤65	33 (52)	
>65	30 (47)	
Gender		0.032
Female	23 (36.5)	
Male	40 (63.5)	
Primary Tumor location		0.033
Colon	40 (64)	
Rectum	23 (36)	
Laterality (Colon)		0.027
Right-sided	13 (33)	
Left-sided	27 (67)	
Preoperative CEA, ng/mL		<0.001
≤5	45 (71)	
>5	18 (29)	
pT		<0.001
T1-T2	16 (25)	
T3-T4	47 (75)	
Nodal metastasis		0.257
No	36 (57)	
Yes	27 (43)	
Tumor Grade		0.432
Low	23 (54)	
Moderate/high	40 (46)	
Lymphatic invasion		0.529
No	34 (54)	
Yes	29 (46)	
Perineural invasion		<0.001
No	47 (75)	
Yes	16 (25)	
TNM stage		0.378
I	12 (19)	
II	23 (36)	
III	20 (32)	
IV	8 (13)	
Chemotherapy		0.131
Neoadjuvant	17 (27)	
Adjuvant	23 (36)	
Both	24 (36)	
MMR status		<0.001
pMMR/MSS	54 (86)	
dMMR/MSI	9 (14)	
Disease recurrence		0.060
No	45 (71)	
Yes	18 (29)	
Survival		0.259
Alive	34 (54)	
Dead	29 (46)	

Abbreviations: CEA, Carcinoembryonic Antigen; pMMR/MSS, mismatch repair proficient/microsatellite stable; dMMR/MSI, mismatch repair deficient/microsatellite instable. Proportion test. Chi-squared test. *p* < 0.05.

**Table 2:** Assdations between fold-change of MREA11, RAD50, NBN, BRCA1, BARD1, RBBP8, PAL B2 gene expression and clinical features of CRC patients.

		MRE11A	RAD50	NBN	BARD1	BRCA1	RBBP8	PALB2
Gender	x2	0.535	0.205	0.034	0.521	0.535	0.476	0.877
	*p*	0.464	0.650	0.853	0.471	0.464	0.490	0.349
Age at diagnosis	x2	1.801	0.005	0.000	3.391	0.067	0.014	5.219
	*p*	0.180	0.942	1.000	0.046a *	0.796	0.904	0.022 *
Tumor site	x2	7.747	1.284	0.548	0.029	0.154	0.128	0.877
	*p*	0.008 *	0.257	0.459	0.865	0.695	0.721	0.349
Sidedness	x2	3.790	2.196	0.005	0.620	0.005	0.014	0.114
	*p*	0.042 *	0.138	0.941	0.431	0.941a	0.906	0.73
CEA	x2	1.802	1.429	0.800	0.022	0.022	1.429	0.089
	*p*	0.148	0.185	0.276	0.560	0.560	0.185	0.489
Tumor size	x2	3.704	0.406	1.429	1.429	1.429	0.229	1.429
	*p*	0.040 *	0.360	0.180	0.180	0.180	0.421	0.180
Histological tumor grade	x2	2.301	0.365	2.191	0.521	0.912	1.471	0.877
	*p*	0.129	0.546	0.139	0.471	0.340	0.225	0.349
Tumor invasive depth	x2	3.298	0.251	5.028	0.220	0.328	2.767	0.017
	*p*	0.039 *	0.616	0.024 *	0.639	0.567	0.096	0.897
Nodal metastasis	x2	0.001	0.022	0.829	2.017	2.872	0.024	9.314
	*p*	0.972	0.882	0.363	0.156a	0.040 *	0.877	0.002 *
TNM stage	x2	0.004	0.000	2.057	4.062	1.322	0.013	10.080
	*p*	0.952	1.000	0.151	0.044 *	0.250	0.910	0.001 *
Lymphovascular invasion	x2	1.435	0.085	0.032	1.137	0.186	0.019	5.907
	*p*	0.231	0.770	0.858	0.286a	0.666	0.891	0.015 *
Perineural invasion	x2	0.961	3.379	0.168	0.220	0.002	0.423	1.516
	*p*	0.610	0.044 *	0.682	0.639	0. 961	0.358	0.173
M SI	x2	3.706	0.770	0.305	0.003	0.305	0.580	2.094
	*p*	0.049 *	0.380	0.581	0.959	0.581	0.446	0.148

Fisher’ s exact test. * Correltion is significant at the 0.05 level (2-tailed). CEA: Carcinoembryonic Antigen; LMR: Lymphocyte-to-monocyte-ratio; NLR: Neutrophil-to-lymphocyte ratio; PLR: Platelet-to-lymphocyte ratio.

**Table 3:** Univariate and multivariate analyses of disease-free survival.

Variable	*p*	Variable	HR (95% CI)	*p*
Age	0.148	<65 years	1.00	0.061
		>65 years	3.79 (0.94–5.32)	
CEA	0.087	<or = 5 ng/mL	1.00	0.299
		>5 ng/mL	3.18 (0.47–11.65)	
Tumor location	0.710			
Sidedness	0.098	Left-sided CRC	1.00	0.500
		Right-sided CRC	2.15 (0.23–10.08)	
Tumor size	0.325			
Tumor grade	0.202			
Lymphovascular invasion	0.029	No	1.00	0.588
		Yes	1.73 (0.24–12.68)	
Perineural invasion	0.206			
TNM stage	0.035	I-II	1.00	0.102
		III-IV	3.75 (0.94–8.54)	
NLR	0.465			
LMR	0.528			
PLR	0.207			
MMR status	0.140	MSS	1.00	0.380
		MSI	2.65 (0.31–3.41)	
*MRE11A*	0.183	Relative low expression	1.00	0.384
		Relative high expression	2.31 (0.35–5.06)	
*RAD50*	0.830			
*NBN*	0.475			
*BARD1*	0.633			
*BRCA1*	0.278			
*RBBP8*	0.636			
*PALB2*	0.269			

Abbreviations: CEA: Carcinoembryonic antigen; LMR: lymphocyte-to-monocyte-ratio; NLR: neutrophil-to-lymphocyte ratio; PLR: platelet-to-lymphocyte ratio.

**Table 4:** Univariate and multivariate analyses of overall survival of CRC patients.

Univariate Analysis		Multivariate Analysis		
Variable	*p*	Variable	HR (95% CI)	*p*
Age	0.306			
CEA	0.803			
Tumor location	0.201			
Sidedness	<0.001	Left-sided	1.00	0.006
		Right-sided	4.57 (1.55–13.49)	
Tumor grade	0.736			
Lymphovascular invasion	0.971			
Perineural invasion	0.970			
TNM stage	0.076	I-II	1.00	0.187
		III-IV	2.55 (0.63–10.30)	
NLR	0.548			
LMR	0.454			
PLR	0.877			
MMR status	0.157	MSS	1.00	
		MSI	0.907 (0.24–3.36)	0.884
*MRE11A*	0.121	Relative low expression	1.00	0.046
		Relative high expression	3.11 (1.64–15.08)	
*RAD50*	0.498			
*NBN*	0.615			
*BARD1*	0.607			
BRCA1	0.608			
*RBBP8*	0.558			
*PALB2*	0.159	Relative low expression	1.00	0.044
		Relative high expression	2.06 (1.67–17.63)	

Abbreviations: CEA: Carcinoembryonic antigen; LMR: lymphocyte-to-monocyte-ratio; NLR: neutrophil-to-lymphocyte ratio; PLR: platelet-to-lymphocyte ratio.

## Data Availability

The datasets generated and analyzed during the current study are not publicly available due to privacy/ethical restrictions but are available from the corresponding authors upon reasonable request. Quantitative RT-PCR was performed using Custom RT2 Profiler PCR Array (#CLAH-32033-9619-6, Qiagen); the primers and sequences were generated using an experimentally verified proprietary computer algorithm and are, therefore, the propriety of QIAGEN.

## Supplementary Materials

The following supporting information can be downloaded at: https://www.mdpi.com/article/10.3390/genes14061270/s1, Figure S1: Representative images of MSI panel by immunohistochemistry; Table S1: Mean fold change of HR gene according to MMR status; Table S2: Mean fold change of HR representative genes according to composite inflammatory blood indexes; Table S3: Associations between inflammatory blood indexes and clinicopathological features of CRC patients.
