# Supplementary material for: High Expression of MRE11A Is Associated with Shorter Survival and a Higher Risk of Death in CRC Patients
Source: Genes (Basel). 2023 Jun 15;14(6):1270. doi: 10.3390/genes14061270 (PMC10298388; doi:10.3390/genes14061270)
Supplement: Supplementary file 1 [file genes-14-01270-s001.zip › Supplementary Figure S1.docx]

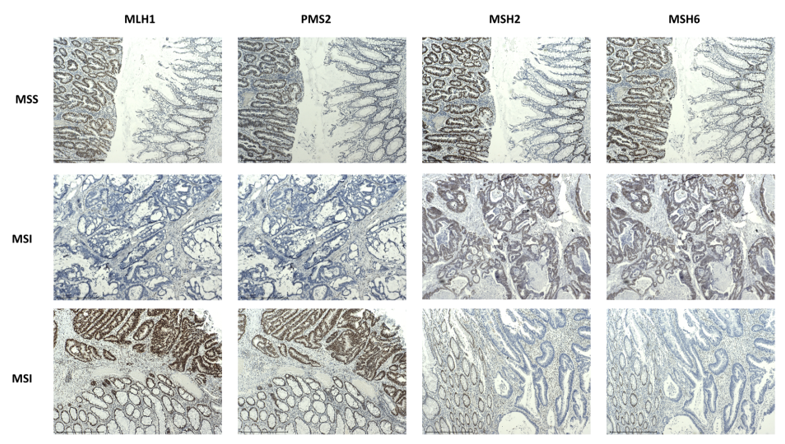


**Supplementary Figure 1.** Representative images of MSI panel by immunohistochemistry. (A) MMR proficient/MSS CRC; MMR deficient/MSI CRC due to loss of (B) MLH1/PMS2 expression or (C) MSH2/MSH6 expression. Original magnification 100×.
