# Supplementary material for: High Expression of MRE11A Is Associated with Shorter Survival and a Higher Risk of Death in CRC Patients
Source: Genes (Basel). 2023 Jun 15;14(6):1270. doi: 10.3390/genes14061270 (PMC10298388; doi:10.3390/genes14061270)
Supplement: Supplementary file 1 [file genes-14-01270-s001.zip › Supplementary Tables S1-S3.docx]

**Supplementary Table 1.** Mean fold-change of HRR gene according to MMR status.

|  | **Mean Difference** | **IC 95%** | ***p*** |
| --- | --- | --- | --- |
| *MREA11* | −2.43 | −4.96–0.10 | **0.005** |
| *RAD50* | −1.82 | −5.32–1.67 | 0.296 |
| *NBN* | −0.57 | −4.18–3.06 | 0.726 |
| *BARD1* | −0.12 | −2.31–2.55 | 0.914 |
| *BRCA1* | −3.07 | −1.12–7.27 | 0.148 |
| *RBBP8* | −1.55 | −3.18–6.29 | 0.512 |
| *PALB2* | −1.14 | −2.18–4.47 | 0.836 |

T-test for mean equality. **p* (2-sided) < 0.05.

**Supplementary Table 2.** Mean fold-change of HRR representative genes according to composite inflammatory blood indexes.

|  |  | ***MRE11A*** | ***P*** | ***RAD50*** | ***p*** | ***NBN*** | ***p*** | ***BARD1*** | ***p*** | ***BRCA1*** | ***p*** | ***RBBP8*** | ***p*** | ***PALB2*** | ***p*** |
| --- | --- | --- | --- | --- | --- | --- | --- | --- | --- | --- | --- | --- | --- | --- | --- |
| **LMR** | Low | 1.49 | 0.720 | −1.39 | 0.239 | 0.11 | **0.039** | 2.02 | 0.141 | 0.81 | 0.574 | −0.44 | 0.292 | 2.34 | 0.746 |
|  | High | 1.85 |  | −2.73 |  | 2.18 |  | 3.18 |  | 1.56 |  | −2.04 |  | 2.71 |  |
| **NLR** | Low | 1.64 | 0.939 | −3.88 | 0.706 | 2.08 | 0.102 | 2.83 | 0.561 | 2.99 | **0.004** | −0.12 | 0.132 | 3.61 | **0.038** |
|  | High | 1.71 |  | −1.85 |  | 0.21 |  | 2.37 |  | -0.67 |  | 3.65 |  | 1.36 |  |
| **PLR** | Low | 1.45 | 0.665 | −2.89 | 0.142 | 2.14 | 0.081 | 3.07 | 0.232 | 1.82 | 0.331 | −1.13 | 0.882 | 2.39 | 0.799 |
|  | High | 1.90 |  | −1.23 |  | 0.15 |  | 2.12 |  | -0.54 |  | −1.36 |  | 2.67 |  |

**Abbreviations:** LMR, Lymphocyte-to-monocyte ratio; NLR, Neutrophil-to-monocyte-ratio; PLR, Platelet-to-lymphocyte ratio. Fold-change means were compared with one-way ANOVA. Mean difference between groups is significant at the 0.05 level (2-tailed) (highlighted in bold).

**Supplementary Table 3.** Associations between inflammatory blood indexes and clinicopathological features of CRC patients.

|  |  | **LMR** | **NLR** | **PLR** |
| --- | --- | --- | --- | --- |
| Gender | χ^2^ | 1.471 | 3.015 | 0.476 |
|  | *p* | 0.297 | 0.070 | 0.603 |
| Age at diagnosis | χ^2^ | **4.573** | 0.014 | 0.014 |
|  | *p* | **0.029 *** | 0.999 | 0.999 |
| Tumor site | χ^2^ | 0.128 | 0.476 | 0.028 |
|  | *p* | 0.462 | 0.603 | 0.998 |
| Sidedness | χ^2^ | 0.014 | 0.631 | 1.026 |
|  | *p* | 0.999 | 0.511 | 0.501 |
| Tumor size | χ^2^ | **3.971** | **6.565** | 2.908 |
|  | *p* | **0.043 *** | **0.021 *** | 0.086 |
| Tumor grade | χ^2^ | 1.471 | 3.015 | 0.028 |
|  | *p* | 0.297 | 0.117 | 0.998 |
| Tumor invasive depth | χ^2^ | 0.255 | **7.960** | 0.235 |
|  | *p* | 0.744 | **0.008 *** | 0.774 |
| Nodal metastasis | χ^2^ | 0.429 | 0.429 | 0.429 |
|  | *p* | 0.613 | 0.613 | 0.613 |
| TNM stage | χ^2^ | 0.384 | 0.384 | 0.013 |
|  | *p* | 0.616 | 0.616 | 0.999 |
| Lymphovascular invasion | χ^2^ | 0.136 | 0.136 | 0.412 |
|  | *p* | 0.802 | 0.802 | 0.616 |
| Perineural invasion | χ^2^ | **3.278** | **3.207** | 0.005 |
|  | *p* | **0.044 *** | **0.040 *** | 1.000 |
| MSI | χ^2^ | 0.648 | 2.439 | 0.002 |
|  | *p* | 0.474 | 0.118 | 1.000 |

χ2 test and Fisher’s exact test. *Correlation is significant at the 0.05 level (2-tailed).
